# Validation of a Machine Learning Approach for Venous Thromboembolism Risk Prediction in Oncology

**DOI:** 10.1155/2017/8781379

**Published:** 2017-09-17

**Authors:** Patrizia Ferroni, Fabio M. Zanzotto, Noemi Scarpato, Silvia Riondino, Fiorella Guadagni, Mario Roselli

**Affiliations:** ^1^Department of Human Sciences and Quality of Life Promotion, San Raffaele Roma Open University, 00166 Rome, Italy; ^2^Interinstitutional Multidisciplinary Biobank (BioBIM), IRCCS San Raffaele Pisana, 00166 Rome, Italy; ^3^Department of Enterprise Engineering, University of Rome “Tor Vergata”, 00133 Rome, Italy; ^4^Department of Systems Medicine, Medical Oncology, University of Rome “Tor Vergata”, 00133 Rome, Italy

## Abstract

Using kernel machine learning (ML) and random optimization (RO) techniques, we recently developed a set of venous thromboembolism (VTE) risk predictors, which could be useful to devise a web interface for VTE risk stratification in chemotherapy-treated cancer patients. This study was designed to validate a model incorporating the two best predictors and to compare their combined performance with that of the currently recommended Khorana score (KS). Age, sex, tumor site/stage, hematological attributes, blood lipids, glycemic indexes, liver and kidney function, BMI, performance status, and supportive and anticancer drugs of 608 cancer outpatients were all entered in the model, with numerical attributes analyzed as continuous values. VTE rate was 7.1%. The VTE risk prediction performance of the combined model resulted in 2.30 positive likelihood ratio (+LR), 0.46 negative LR (−LR), and 4.88 HR (95% CI: 2.54–9.37), with a significant improvement over the KS [HR 1.73 (95% CI: 0.47–6.37)]. These results confirm that a ML approach might be of clinical value for VTE risk stratification in chemotherapy-treated cancer outpatients and suggest that the ML-RO model proposed could be useful to design a web service able to provide physicians with a graphical interface helping in the critical phase of decision making.

## 1. Introduction

In recent years, the grown availability of large sets of electronic health records (big data) has posed new challenging possibilities in terms of data management/analysis, as they exceed the concept of “statistical sampling” in favor of a heuristic search of correlations between phenomena for the construction of predictive models [1].

This is particularly true in oncology, to the point that the 2016 report of the Blue Ribbon Panel of the Cancer Moonshot recommended to mine past patient data for predicting future patient outcomes and for minimizing cancer treatment's debilitating side effects [2].

In this context, a compelling challenge in oncology is predicting the risk of chemotherapy-associated venous thromboembolism (VTE), as VTE occurrence may result in treatment delays, impaired quality of life, and increased mortality [3]. Accordingly, despite thromboprophylaxis for primary prevention is not recommended, assessment of the patient's individual risk of VTE prior to chemotherapy is advocated [4], based on Khorana Score (KS) [5], the sole risk assessment model (RAM) currently available for this clinical setting.

However, even though KS [5] is a user-friendly VTE risk predictor—based on routinely available variables [6]—it is strongly dependent on tumor type and does not consider treatment-related factors influencing VTE development. Therefore, its external validation was not univocal [7–9], its major weakness being represented by a high proportion of patients (>50%) falling into the intermediate risk category [9]. Thus, expanded RAMs including novel biomarkers, potentially improving VTE risk prediction, have been proposed [7]. Yet, their use may be too expensive for widespread screening in low- and middle-income regions.

In this light, we hypothesized that machine learning (ML) would be a solid base to build an inexpensive predictive tool for VTE risk assessment in chemotherapy-treated cancer outpatients that could be easily adapted to different local situations or field advancements [10]. We, therefore, applied a combined approach of kernel ML and random optimization (RO) to design a set of VTE predictors capable of exploiting significant patterns in routinely collected demographic, clinical, and biochemical data that can be used in a clinical decision support system for VTE risk stratification prior to chemotherapy [11]. Among these, we selected the two best predictors out of a range of ten ML-RO runs (ML-RO-2 and ML-RO-3), which could be useful to devise a web-based graphical interface for VTE risk stratification.

Here, we report the results of a monoinstitutional pilot study in which the ML-RO-2 and ML-RO-3 were combined to validate their clinical usefulness in a cohort of 608 ambulatory cancer patients, prospectively followed during chemotherapy at the medical oncology ward of the Tor Vergata Clinical Center.

## 2. Patients and Methods

### 2.1. Patient Dataset

The complete patient dataset for VTE risk assessment (*n* = 1433) was attained by joint efforts between the PTV Bio.Ca.Re. (Policlinico Tor Vergata Biospecimen Cancer Repository) and the BioBIM (Interinstitutional Multidisciplinary Biobank, IRCCS San Raffaele Pisana). The dataset consisted of ambulatory cancer patients in accordance with the principles embodied in the Declaration of Helsinki to investigate possible predictors of chemotherapy-associated VTE. The study was reviewed and approved by the Scientific Institute for Research, Hospitalization and Health Care San Raffaele Pisana and by the Tor Vergata University Institutional Review Boards. All study participants or their legal guardian provided informed written consent about personal and medical data collection prior to study enrollment.

Of the 1433 patients, 825 were included in the original training set used to devise the ML-RO predictors. Clinical characteristics and laboratory attributes of these patients are available at [11]. For the current study, a cohort of 608 patients was attained by implementing the testing set (*n* = 354) analyzed in [11] with patients enrolled thereafter (from July 2015 to June 2016). All patients were chemotherapy naive; specific anticancer treatment was instituted according to international guidelines (11% neoadjuvant, 29% adjuvant, and 60% metastatic; 3% of patients received concurrent radiotherapy). Eligibility criteria were as previously reported [10, 12]. Patients were regularly seen at scheduled visits; additional visits were arranged at the occurrence of clinically suspected VTE. Initial VTE risk stratification was performed by the KS at a 3-point cutoff, as currently recommended [5]. All patients were followed up for a median period of 10 months, during which outcomes were prospectively recorded. The study outcome was defined as the occurrence of a first symptomatic or asymptomatic VTE episode, either deep vein thrombosis (DVT) or pulmonary embolism (PE), during active treatment. No patient received thromboprophylaxis or antiplatelet drugs.

The following variables were taken into consideration: age, sex, tumor site and stage, hematological attributes (including blood cell counts, hemoglobin, and neutrophil- and platelet-lymphocyte ratios), fasting blood lipids [13], glycemic indexes [14], liver and kidney function [15], body mass index (BMI), Eastern Cooperative Oncology Group Performance Status (ECOG-PS), and supportive and anticancer drugs. Numerical attributes were analyzed as continuous values. Variables were clustered into groups according to clinical significance [11].  Table 1 summarizes clinical and laboratory attributes of patients.

### 2.2. Data Analysis

In a context of precision medicine, we introduced a new methodology based on a particular class of learning machines (kernel machines) and on a RO model to devise relative importance of different groups of clinical attributes in the final prediction decisions [11]. The algorithm was devised as previously reported using a 3-fold cross validation technique on a training set. A testing set was used to compute the final performance of our risk predictors. Missing clinical attribute values were treated according to predictive value imputation (PVI) method [16].

A total of 608 patients were entered into the study on the hypothesis that this will detect a difference with a likelihood of >80%, at a two-sided 5% significance level, if the true hazard ratio (HR) is 2. This was based on the assumption of a median follow-up duration of at least 6 months and an estimated VTE rate of 10%. Patients' data are presented as percentages, mean (SD), or median and interquartile range (IQR). Receiver operating characteristic (ROC) curve and Cox proportional hazard analyses were performed by MedCalc Statistical Software version 13.1.2 (MedCalc Software bvba, Ostend, Belgium). Bayesian analysis was performed, and positive (+LR) and negative (−LR) likelihood ratios were used to estimate the probability of having or not having VTE. Survival curves were calculated by the Kaplan-Meier and log-rank methods using a computer software package (Statistica 8.0, StatSoft Inc., Tulsa, OK). VTE-free survival time was calculated from the date of enrollment until the date of VTE (either DVT or PE) or of the last follow-up. For administrative censoring, follow-up was ended at the date of December 20th, 2016. For patients receiving neoadjuvant chemotherapy, follow-up was stopped at completion of an entire antiblastic treatment and before surgery.

## 3. Results and Discussion

No patient underwent surgery during follow-up nor was admitted to a clinic for acute medical illness requiring thromboprophylaxis. VTE was diagnosed in 7.1% of patients (11 PE and 32 DVT; median time to VTE: 2.5 months), and 21 of 43 patients were incidentally diagnosed with asymptomatic VTE (7 PE) at time of CT scan for restaging, in agreement with previous reports [12, 13]. Competing mortality at 6 months was <2%, and 9 patients without VTE died of their disease during this time frame.

**Figure 1:**
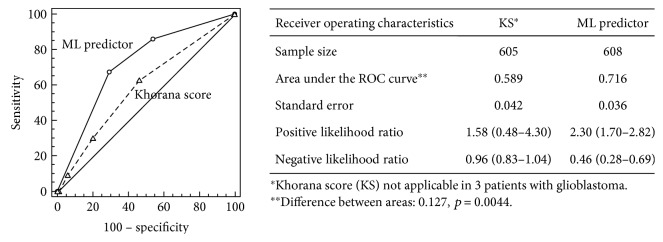
Receiver operating characteristic curves generated from Khorana score (dashed line) and ML-RO VTE predictor (continuous line).

Overall, 37 (6.1%) patients were at high risk for VTE (KS ≥ 3), as per current guidelines. Of these, only 4 (10.8%) patients developed VTE during treatment. On the other hand, 250 (41.5%) patients had an intermediate risk (KS 1 or 2), whereas 318 (52.4%) were classified as low risk based on a KS of 0. VTE rates in the intermediate- and low-risk categories were 9.2% (*n* = 23) and 5.0% (*n* = 16), respectively. Three patients with glioblastoma were not included in the analysis, as the KS is not validated in this cancer type. Accordingly, the overall performance of KS in our population, despite a 94.1% specificity, was characterized by a 9.3% sensitivity, a 10.8% positive predictive value (PPV), and an area under the ROC curve (AUROC) of 0.589, all translating into nonsignificant +LR [1.58 (0.48–4.30)] or −LR [0.96 (0.83–1.04)] (Figure 1). These figures are consistent with the results obtained in the original validation cohort used for the development of KS—showing a high negative predictive value (98.5%), but a PPV lower than 7% [6]—and those by other authors reporting that the majority of events (50% to 85%) occurs in patients at intermediate risk [7, 17, 18]. In this context, it is conceivable to hypothesize that clinical settings, different from that in which KS was originally developed, might be responsible for the inconsistencies observed among various studies.

Undoubtedly, KS represents an interesting endeavor for VTE risk prediction, owing to its ease of use and lack of additional health care costs. However, several reports recently demonstrated that it might not be suitable in specific local situation/populations, such as in the case of lung [9, 19, 20] or pancreatic [21] cancer, where the KS does not correctly stratify patients using a threshold of ≥3 versus <3. An additional explanation of these discrepancies stems from the fact that no information on anticancer [9] or supportive drugs was available for the population used for the development and validation of the KS. Furthermore, Lee and coworkers [19] suggested that the lack of predictive significance of the KS in particular clinical settings could be explained by differences in the proportion of patients with BMI ≥ 35 (e.g., 0.4% in their study versus 12.3% in the one by Khorana et al.), raising the hypothesis that “an area-specific cutoff point for BMI among the Khorana variables should be taken into consideration” in different ethnicities [19].

In this context, the availability of a ML approach that can be locally customized and personalized on individual patient attributes is intriguing. For the present analysis, we selected ML-RO-2 and ML-RO-3 as the best performing risk predictors based on the values of precision [(*P*) positive predictive value in ML], recall [(*R*) sensitivity in ML], and f-measure [a harmonic mean of *P* and *R* calculated as: 2*PR*/(*P* + *R*)] as previously reported [11]. Here, using an extended dataset of 608 patients, both ML-RO-2 and ML-RO-3 showed f-measures of 0.213 and 0.211, respectively, which were substantially higher than that calculated for the KS (f-measure: 0.100) and similar to those originally reported [11], thus confirming the clinical soundness of this approach.

**Figure 2:**
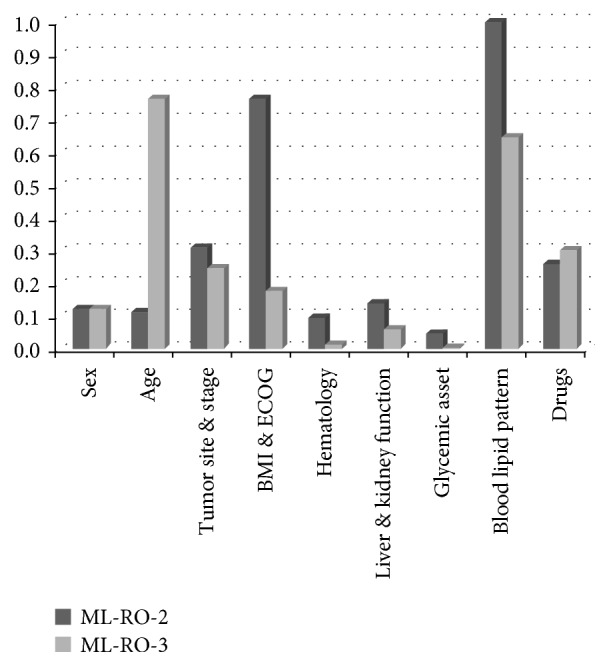
Weights *α*_*i*_ of groups of clinical attributes for the different models [11].

At this point, it is important to emphasize that the two models not only were the best in terms of prediction capacity but they also had a complementary configuration of weights (Figure 2()). In particular, ML-RO-2 was strongly weighted on blood lipids, BMI, and ECOG performance status, while ML-RO-3 had the highest weights for age and blood lipids [11]. This is consistent with literature data showing that low levels of HDL cholesterol [13] and ECOG-PS [22] are among the best predictors of increased VTE risk in chemotherapy-treated cancer patients in multiple regression models. Moreover, tumor site and stage and anticancer drugs maintained a considerable weight in both models (Figure 2()), which is not surprising, since these clinical attributes have also been associated with an increased risk of developing VTE [6, 21, 23].

Nevertheless, the performance of both predictors could be further enhanced. Thus, we sought to investigate whether a combined approach may be of advantage over the individual predictors or the KS. It should be noted that the adoption of a model incorporating a couple of predictors implies that risk evaluation would be represented by a three-level stratification (generated in the event that risk estimate is achieved by both predictors, only one or none of them). However, while this configuration is capable of reducing the number of false negative and false positive, it introduces some degree of uncertainty represented by an intermediate risk class. As reported in Figure 1, the combined model resulted in an overall improvement of VTE risk prediction performance, with a 0.716 AUROC, which was significantly higher than that observed with each single predictor (ML-RO-2 AUROC = 0.680, *p* = 0.05; ML-RO-3 AUROC = 0.670, *p* = 0.02) or KS (difference between areas: 0.127, *p* = 0.0044). At a criterion > 1 (risk estimate achieved by both predictors, according to a voting on the positive class), this combined approach showed a sensitivity (67.4%) and PPV (14.9%) higher than those observed with individual models or KS, resulting in significant +LR [2.30 (1.70–2.82)] and −LR [0.46 (0.28–0.69)].

**Figure 3:**
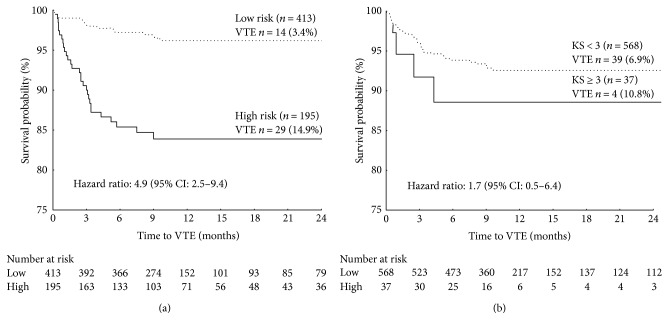
Kaplan-Meier curves of venous thromboembolism- (VTE-) free survival of chemotherapy-treated ambulatory cancer patients in the validation set. Comparison between patients with low (dotted line) or high (solid line) risk of VTE based on ML-RO VTE predictor (a) or Khorana score (b).

**Figure 4:**
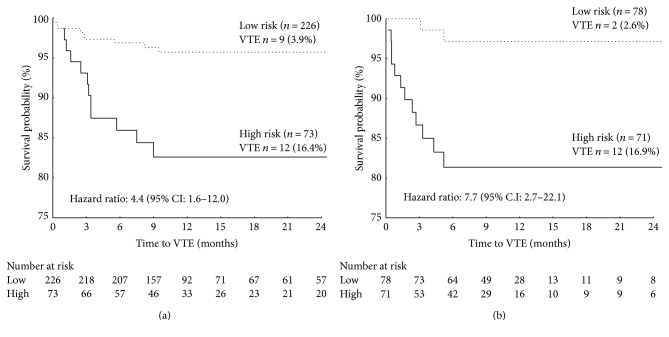
Kaplan-Meier curves of venous thromboembolism- (VTE-) free survival of chemotherapy-treated ambulatory cancer patients categorized by the ML-RO VTE predictor in the validation set. Subgroup analysis of patients with cancer histological types with low ((a) breast and colorectal) or intermediate ((b) lung, gynecologic, and urinary) risk of VTE.

The robustness of this combined model was further corroborated by the results of a VTE-free survival analysis in which patients were considered at risk only in the event of a concordance of both predictors. As shown in Figure 3(a), only 3.4% of patients classified as low risk by the combined ML predictor developed VTE during chemotherapy, compared with 14.9% classified as at risk (log-rank test = 5.29; *p* < 0.0001). On the other hand, despite the high specificity, the KS used at a cutoff ≥ 3 points, as currently recommended, resulted in a 6-month VTE-free survival rate not significantly different from that of low-risk patients (89% versus 94%, resp.; log-rank test = 1.01; *p* = 0.309) (Figure 3(b)). Of interest, the predictive value of the combined ML-RO model was confirmed in a subgroup analysis of patients with tumors generally considered as at low (0 point in the Khorana score) (i.e., breast or colorectal cancers) or intermediate (1 point in the Khorana score) (i.e., lung, gynecologic, or urinary cancers) VTE risk (Figure 4), which further suggest a ML approach may be of advantage over the currently recommended KS.

These results demonstrate that a ML approach, optimizing the relative weight of groups of clinical attributes, might be of clinical value in predicting a first VTE episode in chemotherapy-treated cancer outpatients compared to other RAMs, which are based on the arbitrary assignment of a score according to multivariable analyses.

There are, of course, some limitations to acknowledge. First, the study was monoinstitutional. Second, the sample size was relatively small, ultimately leading to a small number of recorded events. Nonetheless, the data reported here demonstrate that the use of ML algorithms and RO models might be of advantage in developing local classifiers capable of improving VTE risk prediction, while retaining some advantages (e.g., recalculation based on data advance over time) in a perspective of precision medicine. Furthermore, the model proposed here has the unquestionable strength that, since all the variables are usually included in the workout routine of cancer patients, the risk calculation is practically at no cost to the health system. Future application of a ML approach might help oncologists in the difficult phase of decision making, by providing them with the great advantage of limiting observer subjectivity. In particular, the combined use of a set of ML-RO predictors could be useful to design a web service with a graphical interface supporting the oncologist in the critical phase of VTE risk assessment. At present, we are working on the architecture of the decision server and its implementation with the best kernel functions to estimate the risk of VTE on a binary value (at risk and low risk).

## 4. Conclusions

As the world moves toward a big data scenario [24], the possibility to use a machine learning approach to devise a RAM—taking into consideration individual biological variability, environmental exposure, and lifestyle—is particularly appealing and fits well into a context of precision medicine as advocated by the Cancer Moonshot initiative.

## Figures and Tables

**Table 1:** Clinical and laboratory attributes of the patient dataset (*n* = 608).

*Demographics*	
Age, mean ± SD (range)	63 ± 12 (18–88)
*Sex*	
Males	293 (48%)
Females	315 (52%)
BMI, mean ± SD	25.2 ± 4.4

*Primary tumor*	
Colorectal	155 (25%)
Gastric	28 (5%)
Esophageal	10 (2%)
Pancreatic	21 (3%)
Biliary	4 (1%)
Lung	
Non-small cell	81 (13%)
Small cell	15 (3%)
Breast	149 (24%)
Prostate	31 (5%)
Ovarian	16 (3%)
Genitourinary	42 (7%)
Head-neck	23 (4%)
Sarcoma	7 (1%)
Unknown	7 (1%)
Other^∗^	19 (3%)
*Stage of disease*	
Primary	253 (42%)
Relapsing/metastatic	355 (58%)

*Anticancer drugs* ^∗∗^	
Platinum compounds	290 (48%)
Fluoropyrimidine	213 (35%)
Anthracycline	87 (14%)
Taxanes	87 (14%)
Paclitaxel	58 (10%)
Bevacizumab	80 (13.2%)
Gemcitabine	68 (11%)
Irinotecane	79 (13%)
Pemetrexed	38 (6%)
Herceptin	36 (6%)
Antityrosine kinase	16 (3%)
Aromatase inhibitors	60 (10%)
*Supportive drugs, N (%)*	
Erythropoiesis stimulating agents	11 (2%)
Prophylactic myeloid growth factors	18 (3%)
Corticosteroids	109 (18%)

*ECOG-PS, N (%*)	
0	431 (71%)
1	158 (26%)
2	19 (3%)

*Hematology and biochemical attributes*	
Blood cell counts	
Red blood cells	4.5 ± 0.8
Hematocrit	36.6 ± 7.6
Hemoglobin	12.5 ± 1.9
White blood cells	7.7 ± 3.5
Neutrophils	5.2 ± 3.1
Lymphocytes	1.8 ± 1.0
Platelets	261 ± 102
Mean platelet volume	8.6 ± 1.0
Neutrophil-lymphocyte ratio	4.0 ± 4.4
Platelet-lymphocyte ratio	185 ± 145
Routine blood chemistry	
Blood urea nitrogen	38 ± 17
Creatinine	0.9 ± 0.3
eGFR	89.8 ± 28.4
Glucose	110 ± 39
Insulin	28 ± 26
HbA_1c_	6.0 ± 0.9
Total bilirubin	0.6 ± 0.5
Alanine transaminase	24.0 ± 20.0
Aspartate transaminase	25.4 ± 23.1
* γ*-Glutamyl transferase	69 ± 143
Triglycerides	139 ± 82
Total cholesterol	197 ± 52
High-density lipoproteins	48.0 ± 14.1
Low-density lipoproteins	123.1 ± 42.1

*Venous thromboembolism*	
Pulmonary embolism	11 (1.8%)
Deep venous thrombosis	32 (5.3%)
Median time-to-event (months)	2.5 months

BMI: body mass index; ECOG-PS: Eastern Cooperative Oncology Group Performance Status. eGFR: estimated glomerular filtration rate. ^∗^Including mesothelioma (*n* = 4), melanoma (*n* = 3), neuroendocrine tumors (*n* = 3), glioblastoma (*n* = 3), small intestine (*n* = 3), liver (*n* = 2), and one skin cancer. ^∗∗^11% neoadjuvant, 32% adjuvant, and 57% metastatic treatments.
